# Rapid and Ultrasensitive Detection of Methicillin-Resistant *Staphylococcus aureus* Based on CRISPR-Cas12a Combined With Recombinase-Aided Amplification

**DOI:** 10.3389/fmicb.2022.903298

**Published:** 2022-06-03

**Authors:** Ying Wang, Xuan Liang, Jie Xu, Lan Nan, Fang Liu, Guangcai Duan, Haiyan Yang

**Affiliations:** ^1^Department of Epidemiology, College of Public Health, Zhengzhou University, Zhengzhou, China; ^2^Yusuf Hamied Department of Chemistry, University of Cambridge, Cambridge, United Kingdom

**Keywords:** *Staphylococcus aureus*, MRSA, CRISPR-Cas12a, RAA, detection

## Abstract

*Staphylococcus aureus* is one of the main pathogens causing hospital and community-acquired infections, in particular, infections caused by methicillin-resistant *Staphylococcus aureus* (MRSA) cause a higher mortality rate than those caused by methicillin-sensitive strains, which poses a serious global public health problem. Therefore, rapid and ultrasensitive detection of patients with clinical MRSA infection and timely control of infection are essential. Clustered regularly interspaced short palindromic repeats (CRISPR) and CRISPR-associated proteins (Cas) based on nucleic acid detection methods are well-known for its high specificity and sensitivity and programmability. Here, we successfully proposed a method based on CRISPR-Cas12a combined with recombinase-aided amplification (RAA) through fluorescent readout to achieve accurate identification and highly sensitive detection of MRSA in clinical samples. Results showed that the limit of detection (LoD) of the RAA-Cas12a method could reach 10 copies/μl at 60 min of reaction. Specificity tests showed that the method could distinguish MRSA from clinically common bacteria. The results of RAA-Cas12a were consistent with that of antimicrobial susceptibility tests (AST) and polymerase chain reaction (PCR) in 83 clinical samples. These results indicated that the detection method based on RAA-Cas12a has high sensitivity and specificity, and provides important value for rapid detection of MRSA.

## Introduction

*Staphylococcus aureus* (*S. aureus*) is an opportunistic pathogen capable of adapting to different hosts and environmental conditions, and is one of the main causes of various infectious diseases (Antonelli et al., 2019; He and Wunderink, 2020). With the emergence of antibiotics, *S. aureus* infection has been well treated (Lindsay, 2014). However, due to the large-scale and high-frequency use of antibiotics and the impact of various human activities, such as agricultural fertilization and intensive animal feedlots, a major problem associated with *S. aureus* is the significant resistance to various antibiotics, among which methicillin-resistant *S. aureus* (MRSA) is one of the major strains of refractory bacterial infections (Turner et al., 2019). MRSA is defined by the presence of the staphylococcal cassette chromosome *mec* (SCC*mec*) element inserted within the *orfX* gene of *S. aureus* (Ito et al., 2001; Malachowa and DeLeo, 2010). SCC*mec* contains the *mecA* gene complex (responsible for methicillin resistance) and a set of site-specific recombinase genes that are responsible for its mobility (Liu et al., 2016). *mecA* gene encodes an alternative penicillin-binding protein (PBP2a or PBP2′) that has a low affinity for most semisynthetic penicillins, which prevents the β-lactam antibiotics from destroying the bacterial cell wall and thus showing resistance (Pinho et al., 2001; Peacock and Paterson, 2015). The remaining portions of SCC*mec* also carry additional metal and antibiotic resistance genes carried by transposons and plasmids (Miragaia, 2018). SCC*mec* can be frequently transferred between *Staphylococci*, causing more bacteria to develop broad-spectrum resistance (Shore et al., 2011). In addition, among the various MGEs within the MRSA genome, a variety of other toxins that are effective against the human host have been reported (Malachowa and DeLeo, 2010). The gain and loss of virulence determinants carried on MGEs have a vital role in bacterial adaptability, virulence, and survival (Turner et al., 2019). Overall, resistance to widespread antibiotics and carrying a wide variety of virulence factors may make MRSA infections difficult to treat and even have more severe outcomes. Hospital-associated MRSA (HA-MRSA) strains are prevalent in many health care facilities worldwide and are among the most common cause of intravenous-catheter associated infections, ventilator-associated pneumonias, and nosocomial infective endocarditis (Álvarez et al., 2019). Studies have shown that MRSA accounts for 26–60% of clinical infections caused by *S. aureus* (Hu et al., 2016; Di Ruscio et al., 2017). In the meantime, MRSA infection has a higher mortality rate than MSSA (methicillin-sensitive *S. aureus*). Meta-analyses show that MRSA bacteremia is twice as likely to prove fatal as MSSA bacteremia, resulting in longer hospital stays, increased utilization of hospital resources, and a three-fold increase in treatment costs (Lakhundi and Zhang, 2018).

For more effective treatment of MRSA patients, it is very important for clinical workers to select a rapid detection method to accurately and quickly detect patients infected with MRSA. Ideally, it is of great clinical significance to rapidly downgrade from broad-spectrum therapy to targeted antibiotics shortly after or on the same day as patient samples are collected. The most traditional method of bacterial detection in clinical and experimental laboratories is phenotypic methods for antimicrobial resistance detection such as chromogenic media, broth microdilution method (BMD), disk diffusion method, and gradient diffusion methods (Sanchini, 2022). Although these methods can provide accurate identification report, they also have the disadvantages of long culture time (2–4 days), high labor intensity, and being influenced by factors such as culture conditions, inducers, and genetic background. The others are based on molecular methods such as polymerase chain reaction (PCR), nucleic acid sequence-based amplification (NASBA), loop-mediated isothermal amplification (LAMP), and Real-Time quantitative PCR (qPCR) (van Belkum and Rochas, 2018; Chen et al., 2020b; Palavecino, 2020). These methods can effectively improve the detection sensitivity but need professional detection personnel and special equipment.

Recently, great progress has been made in the research on clustered regularly interspaced short palindromic repeats (CRISPR) and CRISPR-associated proteins (Cas) which provides a revolutionary development for nucleic acid molecular diagnosis (Horvath and Barrangou, 2010; Jinek et al., 2012). CRISPR-Cas is an RNA-guided adaptive immune platform for cleaving foreign genetic components of invading viruses and phages (Jinek et al., 2012). An effective CRISPR-Cas system needs two compositions, CRISPR RNAs (crRNAs) or single-guide RNA (sgRNA), and Cas effectors, to form RNP complexes (Jinek et al., 2012). CRISPR systems are classified into two categories based on the composition of Cas effectors. Class 1 systems consist of multiple Cas effectors, while class 2 systems are characterized by only one effector protein. Interestingly, several effectors (Cas12, Cas13, and Cas14) in class 2 systems have not only cis cleavage but also trans cleavage (Swarts and Jinek, 2019). For Cas12a, more specifically, crRNA is employed to recognize the target sequence by a short protospacer-adjacent motif (PAM) sequence (Collias and Beisel, 2021). After the Cas12a-crRNA complex binding with target sequence, the cis cleavage for both strands of double-stranded DNA (dsDNA) target occurs (Chen et al., 2018). And then, non-specific cleavage (trans cleavage) of Cas12a effector was activated to cleave non-specific single-stranded DNA (ssDNA) (Chen et al., 2018). Particularly, the PAM sequence is essential for target sequence recognition but not for non-specific ssDNA (Chen et al., 2018). Based on this property, the CRISPR-Cas system can be developed to detect pathogens, proteins, and biomarkers. At present, SHERLOCK, an RNA targeting method based on Cas13, has been applied to detect viruses such as HBV, ZIKA, and SARS-CoV-2 (Gootenberg et al., 2017; Myhrvold et al., 2018; Joung et al., 2020). This method establishes a complete diagnostic program, which can achieve highly specific and sensitive detection of nucleic acid. However, using Cas13a as an RNase requires a processing step that first converts the amplified DNA into RNA. Instead, Cas12a targets dsDNA and does not require a transcription step (Chen et al., 2018).

**Figure 1:**
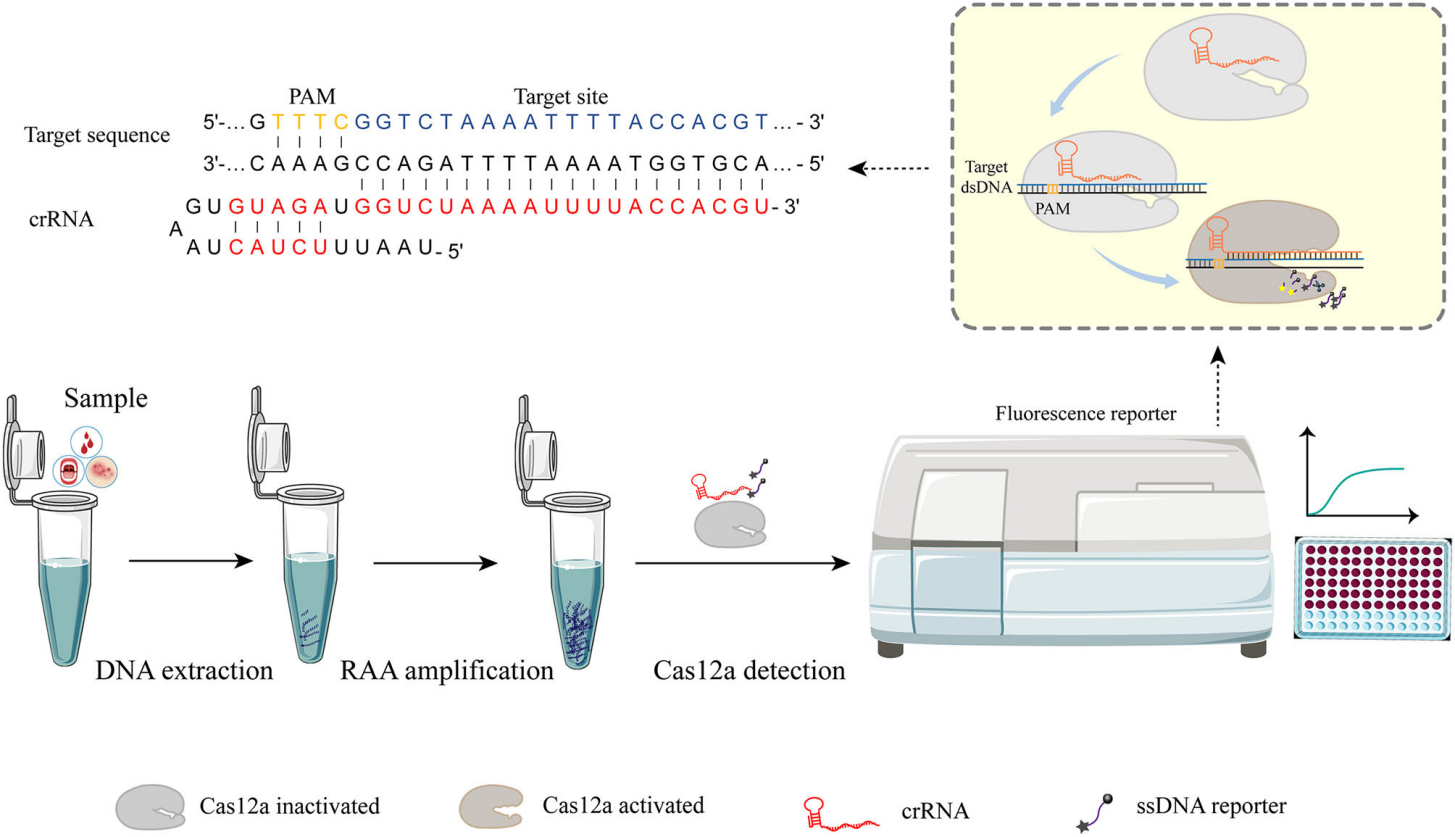
RAA-Cas12a detection system. The detection process can be roughly divided into three parts. Step I, the sample was pretreated and DNA was extracted as the substrate for the next step. Step II, nucleic acid was amplified by recombinase-aided amplification (RAA). Step III, Cas-based signal generation. Cas12a-crRNA complex is activated when crRNA is employed to recognize the target sequence by a short protospacer-adjacent motif (PAM) sequence. After the Cas12a-crRNA complex binding with the target sequence, the cis cleavage for both strands of the dsDNA target occurs. The activated Cas12a-crRNA complex possesses non-specific cleavage (trans cleavage) activity that can cleave non-specific ssDNA reporters to generate fluorescence signals that are captured by the instrument.

Here, we propose a novel MRSA detection strategy that could accurately and sensitively identify MRSA under clinical and experimental conditions. We selected adhesin clumping factor (*clfA*) and thermostable nuclease (*nuc*) genes that have been confirmed by laboratory studies in all *S. aureus* chromosomes as candidates for specific detection of *S. aureus* (Smeltzer et al., 1997; Chen et al., 2020a). Meanwhile, we selected the *mecA* gene as the specific gene of methicillin resistance. The detection process and principle based on RAA-Cas12a are shown in Figure 1. In this study, the MRSA detection method based on RAA-Cas12a is of great significance for the rapid and accurate detection of MRSA infected patients.

## Materials and Methods

### Materials

In this study, all strains were from laboratory preserved strains (Molecular Epidemiology Group, School of Public Health, Zhengzhou University, Zhengzhou, Henan, China), including *S. aureus, Escherichia coli* (*E. coli*), *Staphylococcus epidermidis* (*S. epidermidis*), *Helicobacter pylori* (*H. pylori*), *Shigella sonnei* (*S. sonnei*), *Klebsiella pneumoniae* (*K. pneumoniae*), and *Salmonella typhimurium* (*S. typhimurium*). All synthetic plasmids, DNA fragments [crRNA, primers, and fluorescent probe ssDNA] were synthesized by Sangon Biotech (Shanghai, China). RAA test kit purchased from Hangzhou ZC Bio-Sci&Tech Co. Ltd (Hangzhou, China). CRISPR-Cas12a (Cpf1) (1 μM) was purchased from Bio-lifesci (Guangzhou, China). HiScribe T7 Quick High Yield RNA Synthesis Kit and Monarch® RNA Cleanup Kit were purchased from New England Biolabs (Beijing, China).

### Nucleic Acids Preparation

The strains isolated from the hospital were inoculated on the Columbia blood plate and cultured in 37°C incubator for 18–24 h. Morphological characteristics and hemolysis of the colony were observed. The colonies with morphological characteristics of *Staphylococcus* and hemolytic ring were selected for further identification by Gram staining and slide coagulase test. The DNA extraction used in this paper by bacterial genome DNA extraction kit was purchased from LifeFeng (Shanghai, China). The plasmid was extracted and purified using a plasmid extraction kit (Biomed, Beijing, China).

### crRNA, RPA Primer, and ssDNA Preparation

All *clfA, nuc*, and *mec* (*mecA* and *mecC*) gene sequences of *S. aureus* were obtained from the NCBI database (https://www.ncbi.nlm.nih.gov/) and sequence alignment was performed by using Mega10.1.6 software. Then, conserved sequences matching the PAM of Cas12a (5'-TTTN) were screened, and the corresponding crRNAs were designed as the gRNAs for subsequent detection (Supplementary Table S1). In this process, we did not find a universal crRNA that could simultaneously recognize *mecA* and *mecC*. Therefore, we chose the *mecA* gene, which has a relatively high prevalence, to design the crRNA. To prepare crRNAs, a single-stranded cognate DNA oligonucleotide with a T7 promoter sequence was annealed with a primer sequence having a complementary T7 primer sequence (Supplementary Table S1). It was then transcribed into crRNA using the HiScribe T7 Quick High Yield RNA Synthesis Kit. Subsequently, the product crRNA was purified using Monarch® RNA Cleanup Kit. The optimal crRNA was screened through experiments. Based on the screened optimal crRNA, the corresponding RPA amplification primers and PCR amplification primers were designed by Primer 5 and Oligo 7 software (Supplementary Table S2). Fluorescence probe ssDNA with sequence 5′-6-FAM-TTTT TTTT TTTT-BHQ1 was retrieved in the literature (Ai et al., 2019).

### RAA-Cas12a Detection Method

The whole detection process is divided into two parts: the first part is RAA amplification, and the second part is Cas12a detection. The RAA reaction system was operated strictly according to the instructions. The RAA system consisted of 50 μl: add 38.5 μl A Buffer, 2.0 μl forward primers (10 μM), 2.0 μl reverse primers (10 μM), and 2.0 μl template DNA into the detection unit tube containing the detection dry enzyme preparation, then add 2.5 μl B Buffer into the detection unit tube. Mix thoroughly upside down, centrifuge at low speed for 10 s. The tube was placed in 37°C constant temperature incubators for 30 min to obtain the amplification products. The Cas12a detection system consisted of 50 μl, including 5 μl of RAA amplification products (above products), 5 μl of Cas12a (1 μM), 5 μl of Buffer, 5 μl of fluorescent probe (10 μM), 2.5 μl of crRNA (above crRNA), and 27.5 μl of enzyme-free water. The detection system was placed in a fluorescence reader (microplate reader), and fluorescence intensity was detected with excitation light of 494 nm and emission light of 521 nm, once every 2 min. Continuous fluorescence value reports were obtained at 37°C for 60 min. For the definition of positive results for the fluorescence reader, we set the signal-to-noise ratio parameter (the ratio of the fluorescence value of the detected object to the negative control, S/N) to S/N > 3 after 60 min of Cas12a reaction, which was considered positive results.

### Evaluation of LoD (Limit of Detection)

The synthetic plasmid was obtained by cloning *clfA, nuc, mecA* fragments into pUC57 (Supplementary Table S3) (Liu et al., 2021). After the plasmid was extracted and purified, the concentration was determined using Nanodrop 2000 spectrophotometer (Thermo Scientific, Massachusetts, USA). Copies number was determined following formula copies/μl = (6.02 × 10^23^) × (ng/μl × 10^−9^) / (DNA length × 660) (Ai et al., 2019). The solution was diluted to a concentration of 10^9^-10^0^ copies per μl in TE buffer, from which a 10-fold dilution series was made to serve as the LoD.

### Specificity Determination Experiments

Based on our previous studies and other published literature (Mao et al., 2019; Wei, 2021), we selected bacteria that are more commonly found in clinical infections for specificity experiments. The nucleic acid of *S. aureus, E. coli, S. epidermidis, H. pylori, S. sonnei, K. pneumoniae*, and *S. typhimurium* was extracted, and Cas12a was detected by RAA amplification. Our previous study found that common drug resistance genes in *S. aureus* were *ermC, msrA, tetK, acc(6')-aph(2”), ant(4',4”)*, and *aph(3')-III* (Luo et al., 2018). Strains containing the above resistance genes were selected, and RAA amplification and Cas12a detection were performed using the extracted DNAs as the templates.

### Detection of Clinical Samples

A total of 83 clinical patient samples, including 21 from secretions, 20 from fester, 19 from drainage fluid or indwelling catheters, 14 from sputum, 7 from blood, and 2 from urine, were provided by the hospital in Zhengzhou. Samples were detected by three different methods, including bacterial culture and antimicrobial susceptibility tests (AST), PCR, and RAA-Cas12a detection, among them, using AST as the gold standard, which is commonly used in clinical practice. AST was performed using the broth dilution method according to the recommendations of the Clinical and Laboratory Standards Institute 29th edition (CLSI, 29th ed). The tests were controlled using *S. aureus* ATCC 29213. *S. aureus* with MIC ≥ 4 μg/ml to oxacillin was determined as MRSA. A PCR assay was carried out in a 50 μl reaction mixture, including 25 μl of 2 × Taq PCR Master Mix (purchased from CWBIO, Jiangsu, China), 2 μl of forward primers (10 μM), 2 μl of reverse primers (10 μM), 2 μl of extracted template DNA, and 19 μl of ddH_2_O. A PCR assay was carried out with the following thermal-cycled process: pre-denaturation at 94°C for 2 min, 30 cycles with the following parameters: denaturation at 94°C for 30 s, annealing at 56°C for 30 s, extension at 72°C for 30 s and final extension at 72°C for 5 min. The above experiments were repeated twice to avoid experimental error and ensure the effectiveness of the experiment. S/N > 3 was considered positive results. In addition, the effectiveness of RAA-Cas12a detection was evaluated by sensitivity, specificity, positive predictive value (PPV), negative predictive value (NPV), and Youden's index.

### Statistical Analysis

Data were analyzed using SPSS 21.0 (IBM Crop., Chicago, USA) and GraphPad Prism 8.0.2 (GraphPad Software Inc., Chicago, USA). Fluorescence values were expressed as mean ± SD. The average fluorescence value was obtained by three parallel experiments. Multiple groups were compared with one-way ANOVA method, and differences were considered significant at values of *P* < 0.05.

## Results

### Design and Construction of RAA-Cas12a System

**Figure 2:**
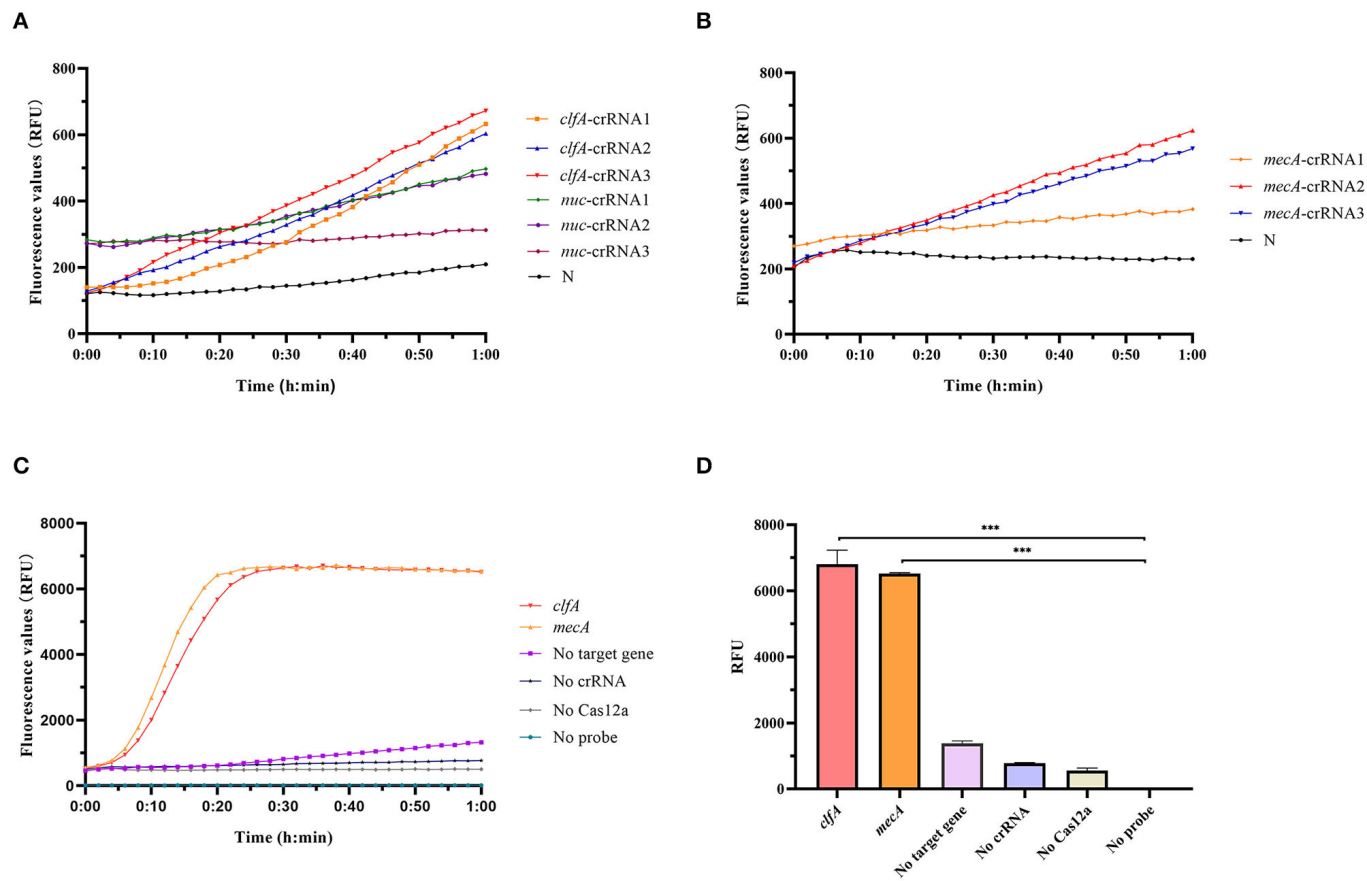
Preparation of Cas12a detection system. **(A)** To screen for optimal crRNA that specifically identify *S. aureus*, we designed different crRNAs for *nuc* and *clfA* genes and simultaneously performed Cas12a detection for *S. aureus* with the same concentration without amplification. **(B)** Different crRNAs were designed for the *mecA* gene, and Cas12a detection was performed on unamplified MRSA DNA to screen out crRNA with better specificity to recognize MRSA. **(C)** Cas12a detection system fluorescence report curve. **(D)** Comparison of fluorescence values generated after 60 min of Cas12a reaction, ***p < 0.0001. Data were expressed as mean ± SD (*n* = 3).

Cas12a detection system was used to detect clinical MRSA strains, and sequence alignment of *clfA, nuc*, and *mecA* sequences was conducted to screen out conserved target regions, and different crRNAs were designed (Supplementary Table S1). Then, we constructed a system, consisting of Cas12a, Buffer, probe, MRSA genomic DNA, and different crRNAs designed, to screen the optimal crRNA. Our experimental results showed that *clfA*-crRNA3 and *mecA*-crRNA2 combined with Cas12a had a better shear effect on ssDNA (Figures 2A,B). Therefore, *clfA*-crRNA3 and *mecA*-crRNA2 were selected for subsequent experiments.

As shown in Figures 2C,D, we successfully constructed the RAA-Cas12a detection system. Only if the Cas12a protein, crRNA, probe, and target gene were present in one system simultaneously, can the Cas12a protein bind the crRNA and target gene to form a complex that plays the role of shearing the ssDNA probe, thus producing a fluorescent signal that is captured by the instrument.

### Evaluation of LoD of RAA-Cas12a Detection System

**Figure 3:**
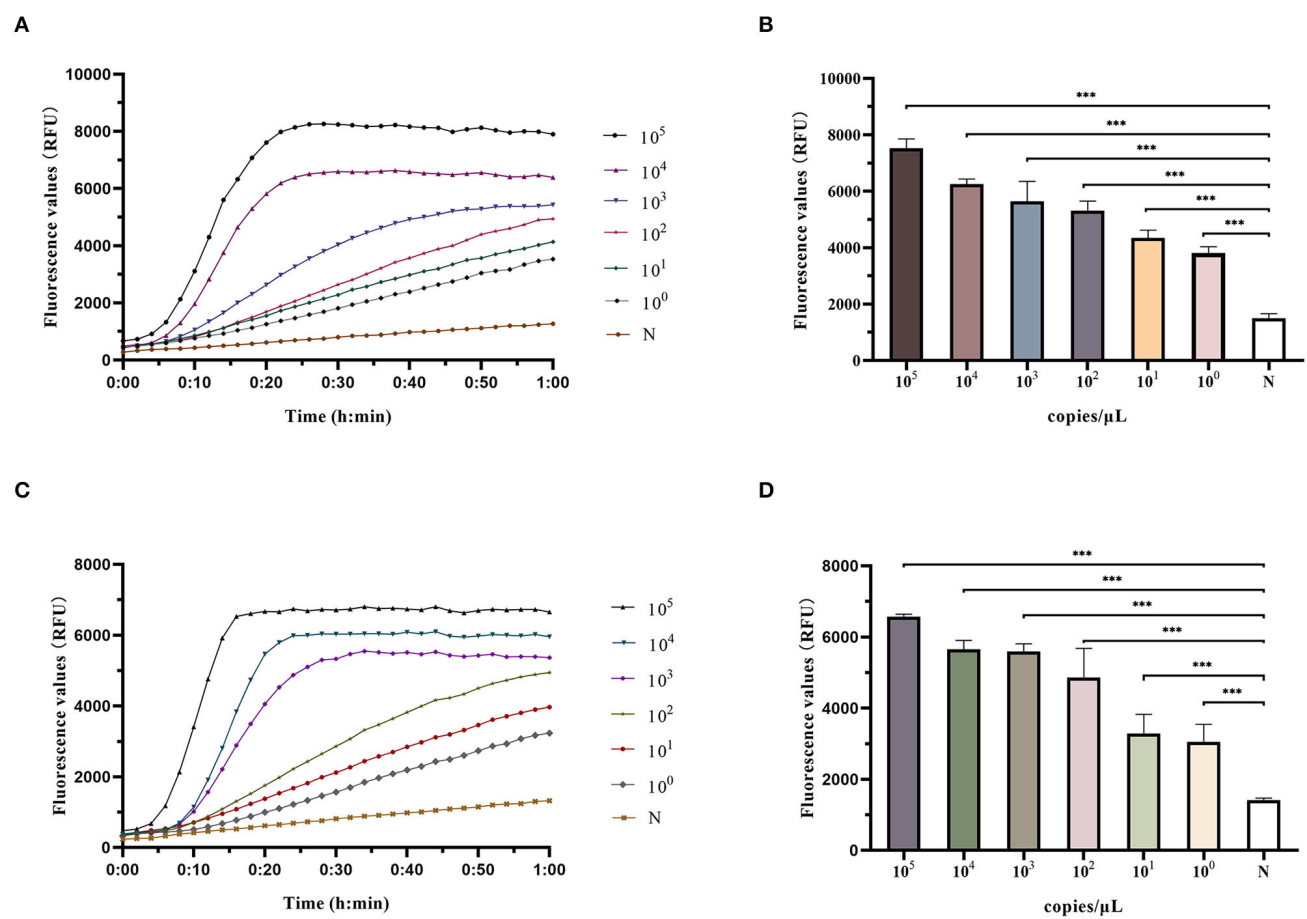
Determination of LoD (limit of detection) of RAA-Cas12a detection system. Data were expressed as mean ± SD (*n* = 3). Negative control (N) utilized RNase-free water as input instead of DNA dilutions. ***p < 0.0001. **(A)** Fluorescence curves were generated when Cas12a reacts with the *clfA* target at each dilution. **(B)** Comparison of fluorescence values generated after 60 min of Cas12a reaction (*clfA* target). **(C)** Fluorescence curves were generated when Cas12a reacts with the *mecA* target at each dilution. **(D)** Comparison of fluorescence values generated after 60 min of Cas12a reaction (*mecA* target).

A series of gradient dilutions of the synthetic plasmid containing *clfA* as a template was amplified using RAA for 30 min and detected using the Cas12a system. The results showed that the fluorescence intensity of the products at 10^5^ and 10^4^ copies/μl concentration reached its peak within 30 min of incubation at 37°C. The fluorescence intensity at 10^3^-10^0^ copies/μl concentration was significantly higher than that in the negative control group, and the difference was statistically significant (*P* < 0.0001; Figures 3A,B). The RAA-Cas12a system was used to detect synthetic plasmids containing *mecA* using the same method. The results showed that the fluorescence intensity at 10^5^-10^3^ copies/μl concentration reached the peak within 30 min of incubation at 37°C, and the fluorescence intensity at 10^2^ to 10^0^ copies/μl concentration was significantly higher than that of the negative control group, the difference was statistically significant (*P* < 0.0001; Figures 3C,D). However, the results of the fluorescence values at 10^0^ copy/μl did not meet our predefined criteria for positive results S/N > 3. Therefore, the LoD of this detection method could reach 10 copies/μl at 60 min of reaction.

After gradient dilution of the synthetic plasmid containing *clfA* gene, PCR amplification was performed, and the results of agarose gel electrophoresis experiments showed there was no obvious band when the concentration was below 10^5^ copies/μl (Supplementary Figures S1, S2). This indicates that RAA-Cas12a method had higher sensitivity than PCR method.

### Specificity of RAA-Cas12a Detection System

**Figure 4:**
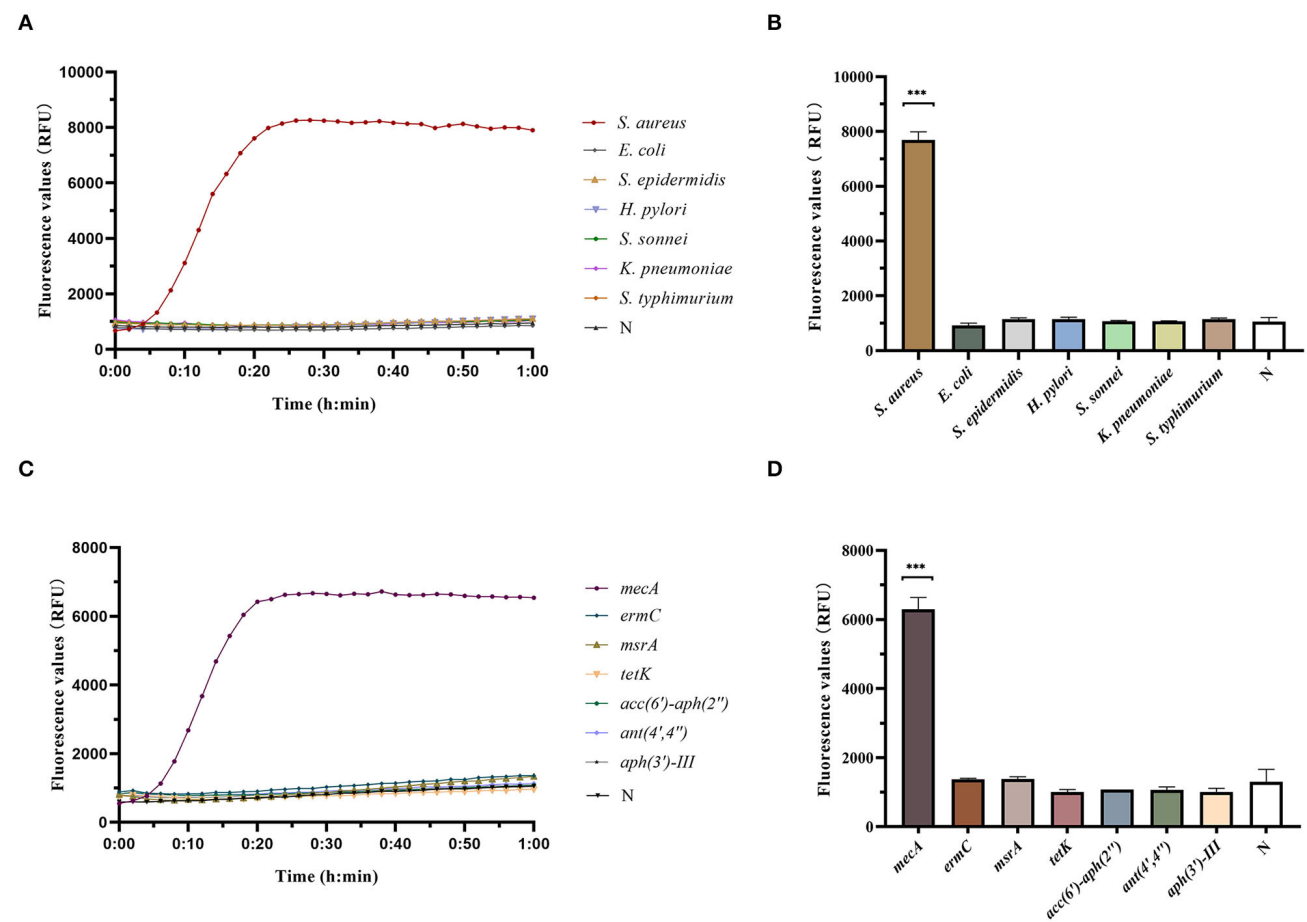
Determination of specificity of RAA-Cas12a detection system. Data were expressed as mean ± SD (*n* = 3). Negative control (N) utilized RNase-free water as input instead of DNA dilutions. ***p < 0.0001. **(A)** Fluorescence curves were generated when different clinical strain samples were detected by the RAA-Cas12a detection system. **(B)** Cas12a reaction was performed after 60 min to compare the fluorescence values of different clinical strain samples. **(C)** Fluorescence curves were generated when different antibiotic resistance genes were detected by the RAA-Cas12a detection system. **(D)** Cas12a reaction was performed after 60 min to compare the fluorescence values of different antibiotic resistance genes.

Different strains were detected by RAA-Cas12a, and the results showed that only *S. aureus* had an amplification curve after detection. There were no amplification curves for *E. coli, S. epidermidis, H. pylori, S. sonnei, K. pneumoniae*, and *S. typhimurium*, indicating that the RAA-Cas12a detection system had high specificity for *S. aureus* (Figures 4A,B).

RAA-Cas12a was used to detect common resistance genes in bacteria, and the results showed that only the strains containing *mecA* had amplification curves. Whereas, the strains, containing *ermC, msrA, tetK, acc(6')-aph(2”), ant(4',4”)* and/or *aph(3')-III*, had no amplification curve, indicating that the RAA-Cas12a detection system had high specificity for *mecA* (Figures 4C,D).

### Clinical Samples Detection by the RAA-Cas12a System

**Figure 5:**
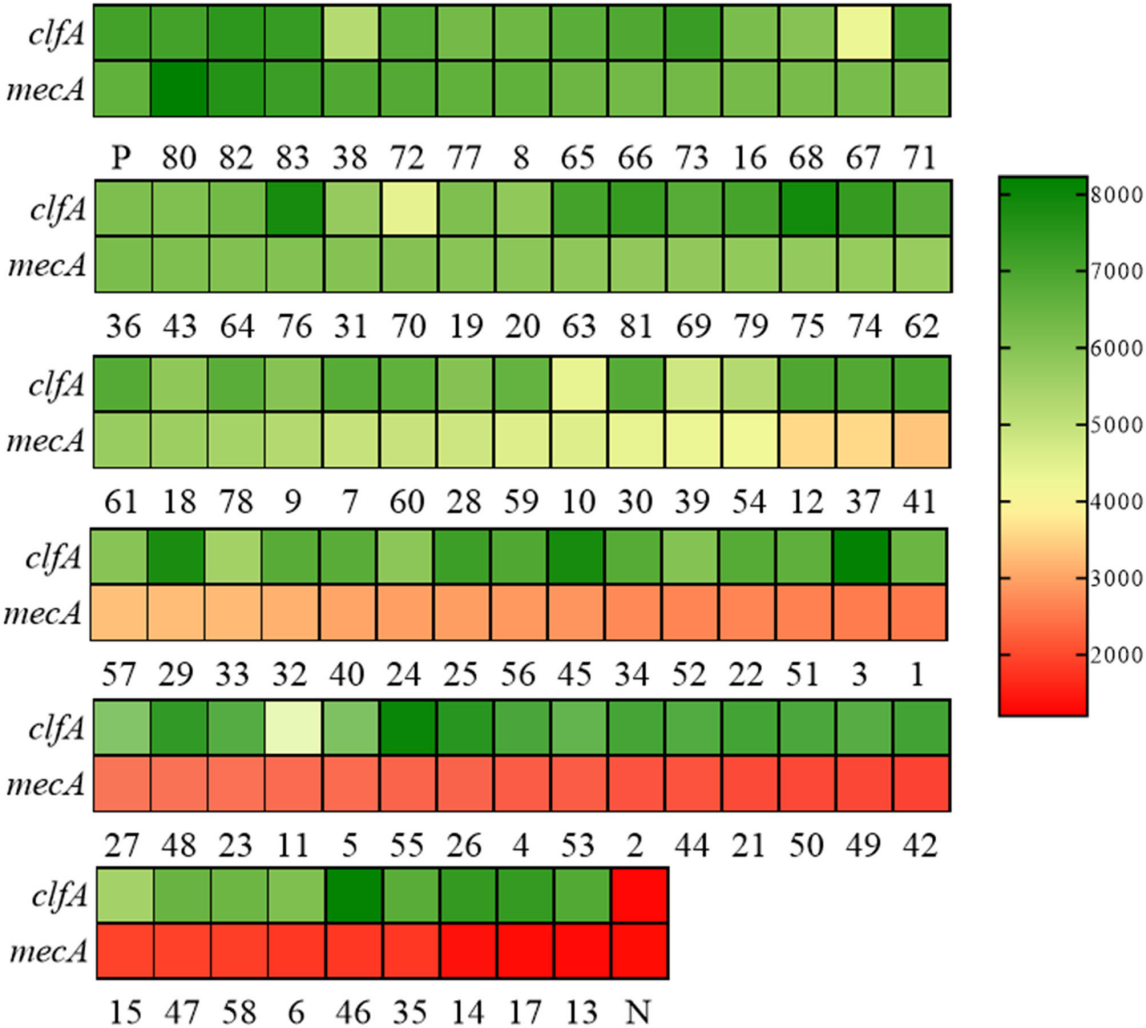
RAA-Cas12a detection system for MRSA in 83 clinical samples. Green indicates that the gene detected in the sample is positive, and the shade of the color is positively correlated with the fluorescence value. Red indicates that the gene tested in the sample is negative, and the depth of red is negatively correlated with the fluorescence value. Positive control (P) and negative control (N). Both *clfA* and *mecA* tests are positive, indicating that the sample is MRSA.

To evaluate the effectiveness of the RAA-Cas12a detection system in clinical samples, 83 clinically isolated *S. aureus* strains were assayed using AST, PCR, and RAA-Cas12a detection system. The results of AST are shown in Supplementary Table S4, we found that 41 isolates were phenotypically classed as MRSA. And then PCR method was used to detect *mecA* of these 83 strains (Supplementary Figure S3). The results of agarose gel electrophoresis experiments showed that 41 strains of *S. aureus* contained the 500 bp gene. The amplified products were sent for sequencing, and the BLAST sequence indicated that the gene amplified was *mecA*, proving that all 41 strains were MRSA. At the same time, these 83 strains were detected by the RAA-Cas12a detection system. All 83 strains were *S. aureus*, among which 41 strains of MRSA samples were detected (Figure 5). The results showed that MRSA detection based on RAA-Cas12a was consistent with AST and PCR. The results of sensitivity, specificity, PPA, NPA, and Youden's index are shown in Table 1, indicating that this method was fully comparable with AST and PCR.

**Table 1:** RAA-Cas12a, AST and PCR for clinical samples detection.

		**AST**		**Total**	**Sensitivity**	**Specificity**	**PPA**	**NPA**	**YI**
		**MRSA**	**MSSA**		**(95% CI)**	**(95% CI)**	**(95% CI)**	**(95% CI)**	**(95% CI)**
PCR	MRSA	41	0	41	100%	100%	100%	100%	100%
	MSSA	0	42	42	(89.3–100%)	(89.6–100%)	(89.3–100%)	(89.6–100%)	(78.9–100%)
	Total	41	42	83					
RAA-Cas12a	MRSA	41	0	41	100%	100%	100%	100%	100%
	MSSA	0	42	42	(89.3–100%)	(89.6–100%)	(89.3–100%)	(89.6–100%)	(78.9–100%)
	Total	41	42	83					

*AST, antimicrobial susceptibility tests; PCR, polymerase chain reaction; MRSA, methicillin-resistant Staphylococcus aureus; MSSA, methicillin-sensitive Staphylococcus aureus; RAA, recombinase-aided amplification; PPA, positive predictive agreement; NPA, negative predictive agreement; YI, Youden's index. The gold standard for MRSA is AST*.

## Discussion

MRSA, due to its wide antibiotic resistance spectrum, fast transmission speed, and easy to cause outbreaks, is one of the typical representatives of multi-drug resistant bacteria (Lindsay, 2013). Therefore, the sensitive, specific, and rapid detection of MRSA strains is the key from broad-spectrum antibiotic treatment to specific antibiotic treatment. As we know, genetic information is contained in the nucleotide sequence of DNA (and sometimes RNA), therefore, in molecular diagnostics, the detection of DNA is crucial for the identification of microorganisms of interest, and PCR is a mature nucleic acid amplification technology (Matsuda, 2017). It has been applied to microbial detection in a variety of fields, including clinical, food, and environmental. However, the PCR-based methods are faced with some inevitable shortcomings, such as complex trained personnel, thermal cycling procedures, the accuracy of reaction steps, and the bulky experimental equipment (Maurer, 2011; Chen et al., 2019; Qian et al., 2022). Compared to PCR, the RAA technique is an alternative method that uses different mechanisms throughout the reaction process at a constant temperature and does not require complex thermal cycles, making it ideal for point-of-care detection (Fan et al., 2020). In this study, gel electrophoresis results after PCR amplification showed that the *mecA* gene could not be observed at the dilution concentration of 10^5^ copies/μl. However, the detection results of the RAA-Cas12a system showed that the LoD of this detection method could reach 10 copies/μl at 60 min of reaction. These results indicate that the established RAA-Cas12a method has a high sensitivity. In addition, because both RAA amplification and Cas12a reactions can function at 37°C, a complex thermal cycling process is avoided, a small incubator or water bath can meet the testing needs, and can be used in primary health care institutions.

In recent years, emerging nucleic acid detection technologies based on CRISPR-Cas developments have opened up new opportunities for pathogen detection (Peng et al., 2020; Zhou et al., 2020; Yin et al., 2021). Compared to traditional assays, the CRISPR-Cas system has low requirements on sample quality and strong anti-interference ability and can be combined with rapid sample pretreatment technology to extract nucleic acids in the field without relying on professional equipment (Ding et al., 2021a). It has been shown that a visualized and rapid detection technology of CRISPR-Cas13a nucleic acid suitable for the lateral flow test strip technology was established by modifying biotin and small molecule antigen FAM at both ends of the reporter RNA (Gootenberg et al., 2018; Ding et al., 2021b). Gootenberg et al. have successfully applied the technology to rapid tests for ZIKA (Gootenberg et al., 2018). Compared with traditional methods, the lateral flow test strip technology is portable and fast, enabling rapid and visual detection with the naked eye in the field. The detection system can realize convenient visual detection of specific nucleic acid (pathogen, gene mutation, etc.). However, the sensitivity of the lateral flow test strip technology based on the RAA-Cas12a system for MRSA detection in this study is not particularly ideal. After the development and improvement of lateral flow test strip technology, RAA-Cas12a technology can be transformed into a convenient visual test paper, which can further simplify the detection process and reduce the detection time (Arizti-Sanz et al., 2020).

Although the RAA-Cas12a method we established has high sensitivity and specificity, it still has several weaknesses. First, our method does not detect the few MRSA strains containing the *mecA*-like gene (*mecC*) (Lakhundi and Zhang, 2018), which has also been observed in other molecular strategies (Buchan et al., 2015; Xu et al., 2020; Suea-Ngam et al., 2021). *mecC* is a new resistance gene recently discovered and identified in MRSA isolated from animals. Although *mecA*- and *mecC*-encoded proteins possess different biochemical properties, *mecC* nevertheless confers methicillin resistance (García-Garrote et al., 2014). Because the Cas effector's recognition requirements for PAM lead to the narrowing of the range of recognizable sequences, we did not find a target sequence for simultaneous recognition of *mec*. Second, due to the limitations of current basic research, we only used Cas12 for detection. Gootenberg et al. revealed that different Cas effectors with unique cleavage preferences for different dinucleotide motifs, and multiple sensing of different targets can be achieved (Gootenberg et al., 2018). However, multiplex detection is still a challenge for CRISPR-Cas (Li et al., 2019), with the development of CRISPR-Cas technology, the functions of Cas effectors have become clearer, and in the coming years it is possible to combine different Cas effectors simultaneously for purpose of simultaneous detection of different target genes.

## Conclusion

In this study, we successfully constructed the CRISPR-Cas12a combined with the RAA method based on fluorescence readout, realizing the accurate identification and high sensitivity detection of MRSA in clinical samples. The results of RAA-Cas12a were consistent with AST and PCR results of 83 clinical samples. Although the clinical application of the CRISPR-Cas system is still in its infancy and has some shortcomings, its emergence opens up many possibilities for the biomedical field. With the deepening of research and clinical verification, we believe that the application of the CRISPR-Cas system will play an irreplaceable role in pathogen detection, drug resistance gene detection, and other aspects.

## Data Availability Statement

The original contributions presented in the study are included in the article/Supplementary Material, further inquiries can be directed to the corresponding author.

## Ethics Statement

The use of clinical samples has been reviewed by the Life Sciences Ethics Review Committee of Zhengzhou University (ZZUIRB 2019-003).

## Author Contributions

YW designed this study. YW and XL finished the experiments and wrote the manuscripts together. YW, XL, JX, LN, FL, GD, and HY revised the manuscript critically for important intellectual content. All authors read and approved the final manuscript.

## Funding

This work was funded by the National Natural Science Foundation of China (No. 81973105). The funder had no role in the study design, data collection, and analysis, decision to publish, or preparation of the manuscript.

## Conflict of Interest

The authors declare that the research was conducted in the absence of any commercial or financial relationships that could be construed as a potential conflict of interest.

## Publisher's Note

All claims expressed in this article are solely those of the authors and do not necessarily represent those of their affiliated organizations, or those of the publisher, the editors and the reviewers. Any product that may be evaluated in this article, or claim that may be made by its manufacturer, is not guaranteed or endorsed by the publisher.
