# Supplementary material for: Rapid and Ultrasensitive Detection of Methicillin-Resistant Staphylococcus aureus Based on CRISPR-Cas12a Combined With Recombinase-Aided Amplification
Source: Front Microbiol. 2022 Jun 3;13:903298. doi: 10.3389/fmicb.2022.903298 (PMC9204182; doi:10.3389/fmicb.2022.903298)
Supplement: Supplementary file 1 [file Data_Sheet_1.docx]

Table S1. crRNA sequences

| crRNA name | Sequences (5′–3′) |
| --- | --- |
| *clfA*-crRNA1 | UAAUUUCUACUAAGUGUAGAUUCAAUUAAAGGUACGAUUGA |
| *clfA*-crRNA2 | UAAUUUCUACUAAGUGUAGAUUGUCAAUCCAAGCGGAGAUA |
| *clfA*-crRNA3 | UAAUUUCUACUAAGUGUAGAUGGACUACUCAGCAGUAAAGA |
| *nuc*-crRNA1 | UAAUUUCUACUAAGUGUAGAUUCUACACCUUUUUUAGGAUG |
| *nuc*-crRNA2 | UAAUUUCUACUAAGUGUAGAUUACAUUAAUUUAACCGUAUC |
| *nuc*-crRNA3 | UAAUUUCUACUAAGUGUAGAUUGUAAUUUUUUAGUUGAAGUU |
| *mecA*-crRNA1 | UAAUUUCUACUAAGUGUAGAUUUAUAUUCUUCGUUACUCAU |
| *mecA*-crRNA2 | UAAUUUCUACUAAGUGUAGAUGGUCUAAAAUUUUACCACGU |
| *mecA*-crRNA3 | UAAUUUCUACUAAGUGUAGAUUCGGACGUUCAGUCAUUUCU |
| T7 primer | TAATACGACTCACTATAGGG |
| Template *clfA*-crRNA1 | TCAATCGTACCTTTAATTGAATCTACACTTAGTAGAAATTACCCTATAGTGAGTCGTATTA |
| Template *clfA*-crRNA2 | TATCTCCGCTTGGATTGACAATCTACACTTAGTAGAAATTACCCTATAGTGAGTCGTATTA |
| Template *clfA*-crRNA3 | TCTTTACTGCTGAGTAGTCCATCTACACTTAGTAGAAATTACCCTATAGTGAGTCGTATTA |
| Template *nuc*-crRNA1 | CATCCTAAAAAAGGTGTAGAATCTACACTTAGTAGAAATTACCCTATAGTGAGTCGTATTA |
| Template *nuc*-crRNA2 | GATACGGTTAAATTAATGTAATCTACACTTAGTAGAAATTACCCTATAGTGAGTCGTATTA |
| Template *nuc*-crRNA3 | AACTTCAACTAAAAAATTACAATCTACACTTAGTAGAAATTACCCTATAGTGAGTCGTATTA |
| Template *mecA*-crRNA1 | ATGAGTAACGAAGAATATAAATCTACACTTAGTAGAAATTACCCTATAGTGAGTCGTATTA |
| Template *mecA*-crRNA2 | ACGTGGTAAAATTTTAGACCATCTACACTTAGTAGAAATTACCCTATAGTGAGTCGTATTA |
| Template *mecA*-crRNA3 | AGAAATGACTGAACGTCCGAATCTACACTTAGTAGAAATTACCCTATAGTGAGTCGTATTA |

Table S2. Primers sequences

| Primer name | Sequences (5′–3′) | Amplicon length (bp) |
| --- | --- | --- |
| RAA-*clfA*-3-F | ATGAATATGAAGAAAAAAGAAAAACACGCAATTC | 185 |
| RAA-*clfA*-3-R | ACGCTACTTGAATCATTACTTTTGCTTTCGTTAC |  |
| RAA-*mecA*-2-F | CATTTGTTGTTTGATATAGTCTTCAGAAATACTTAGT | 200 |
| RAA-*mecA*-2-R | GAATGCAGAAAGACCAAAGCATACATATTGAAAA |  |
| PCR-*clfA*-3-F | AGAAAAACACGCAATTCGGAA | 175 |
| PCR-*clfA*-3-R | GCACTAACGCTACTTGAATCATT |  |
| PCR- *mecA*-2-F | TCTTTGGAACGATGCCTATCT | 153 |
| PCR- *mecA*-2-R | GGGATCATAGCGTCATTATTCC |  |
| PCR- *mecA*-F | CACCTTGTCCGTAACCTGAA | 536 |
| PCR- *mecA*-R | TGGCTCAGGTACTGCTATCC |  |

Table S3. Reference plasmid sequences

| Plasmid name | Sequences (5′–3′) | Length (bp) |
| --- | --- | --- |
| Reference plasmids containing *clfA* | ATGAATATGAAGAAAAAAGAAAAACACGCAATTCGGAAAAAATCGATTGGCGTGGCTTCAGTGCTTGTAGGTACGTTAATCGGTTTTGGACTACTCAGCAGTAAAGAAGCAGATGCAAGTGAAAATAGTGTTACGCAATCTGATAGCGCAAGTAACGAAAGCAAAAGTAATGATTCAAGTAGCGTTAGTGCTGCACCTAAAACAGACGACACAAACGTGAGTGATACTAAAACATCGTCAAACACTAATAATGGCGAAACGAGTGTGGCGCAAAATCCAGCACAACAGGAAACGACACAATCATCATCAACAAATGCAACTACGGAAGAAACGCCGGTAACTGGTGAAGCTACTACTACGACAACGAATCAAGCTAATACACCGGCAACAACTCAATCAAGCAATACAAATGCGGAGGAATTAGTGAATCAAACAAGTAATGAAACGACTTCTAATGATACTAATACAGTATCATCTGTAAATTCACCTCAAAATTCTACAAATGCGGAAAATGTTTCAACAACGCAAGATACTTCAACTGAAGCAACACCTTCAAACAATGAATCAGCTCCACAGAATACAGATGCAAGTAATAAAGAT | 600 |
| Reference plasmids containing *nuc* | TTATTGACCTGAATCAGCGTTGTCTTCGCTCCAAATATTTAATTTCTCTTTTTTCGCTTGTGCTTCACTTTTTCTTAAAAGTTGTTCATGTGTATTGTTAGGTTTATAAACATAAGCAACTTTAGCCAAGCCTTGACGAACTAAAGCTTCGTTTACCATTTTTCCATCAGCATAAATATACGCTAAGCCACGTCCATATTTATCAGTTCTTTGACCTTTGTCAAACTCGACTTCAATTTTATTTGCATTTTCTACCATTTTTTTCGTAAATGCACTTGCTTCAGGGCCATATTTCTCTACACCTTTTTTAGGATGCTTTGTTTCAGGTGTATCAACTAATAATAGTCTGAATGTCATTGGTTGACCTTTGTACATTAATTTAACCGTATCACCATCAATCGCTTTAATTAATGTCGCAGGTTCTTTATGTAATTTTTTAGTTGAAGTTGCACTATATACTGTTGGATCTTCAGAACCACTTCTATTTACGCCGTTATCTGTTTGTGATGCATTTGCTGAGCTACTTAGACTTGAAACTACAACTAAAGTTAACACTAAGCAACTAGTAGCGAAAAAGAAAAACCTCTTTGCGTATTGCCCTTTCGAAACATTACTGATAGCCATCCCTATAAGTAATATTGAAACAATTGCCATACATATGCCAGCACTTAATAAGTATTCTGTCAT | 687 |
| Reference plasmids containing *mecA* | ACCAACATAACCTAATAGATGTGAAGTCGCTTTTTCTAGAGGATAGTTACGACTTTTTGTTTCATTAGTTGTAAGATGAAATTTTTTTGCGAAATCACTTAAATATTCATCCATTTTTTTAACGGTTTTAAGTGGAACGAAGGTATCATCTTGTACCCAATTTTGATCCATTTGTTGTTTGATATAGTCTTCAGAAATACTTAGTTCTTTAGCGATTGCTTTATAATCTTTTTTAGATACATTCTTTGGAACGATGCCTATCTCATATGCTGTTCCTGTATTGGCCAATTCCACATTGTTTCGGTCTAAAATTTTACCACGTTCTGATTTTAAATTTTCAATATGTATGCTTTGGTCTTTCTGCATTCCTGGAATAATGACGCTATGATCCCAATCTAACTTCCACATACCATCTTCTTTAACAAAATTAAATTGAACGTTGCGATCAATGTTACCGTAGTTTGTTTTAATTTTATATTGAGCATCTACTCGTTTTTTATTTTTAGATACTTTTTTTATTTTACGATCCTGAATGTTTATATCTTTAACGCCTAAACTATTATATATTTTTATCGGACGTTCAGTCATTTCTACTTCACCATTATCGCTTTTAGAAATATAACTGCTATCTTTATAAACTTGTTTGAAATTTTTATCTTCAATTGCATCAATAGTATTATTAATTTCTTTATCTTTTGAAGCATAAAAATATATACCAAACCCGACAACTACAACTATTAAAATAAGTGGAACAATTTTTATCTTTTTCAT | 811 |
|  |  |  |

Table S4. Drug resistance rate of *Staphylococcus aureus* of clinical specimen

| Antibiotics | The number of drug-resistant (n) | Drug resistance rate (%) |
| --- | --- | --- |
| Penicillin | 80 | 96.39 |
| Erythromycin | 72 | 86.75 |
| Oxacillin | 41 | 49.40 |
| Tetracycline | 38 | 45.78 |
| Ciprofloxacin | 26 | 31.33 |
| Gentamicin | 23 | 27.71 |
| Rifampicin | 7 | 8.43 |
| Vancomycin | 0 | 0 |
| Linezolid | 0 | 0 |


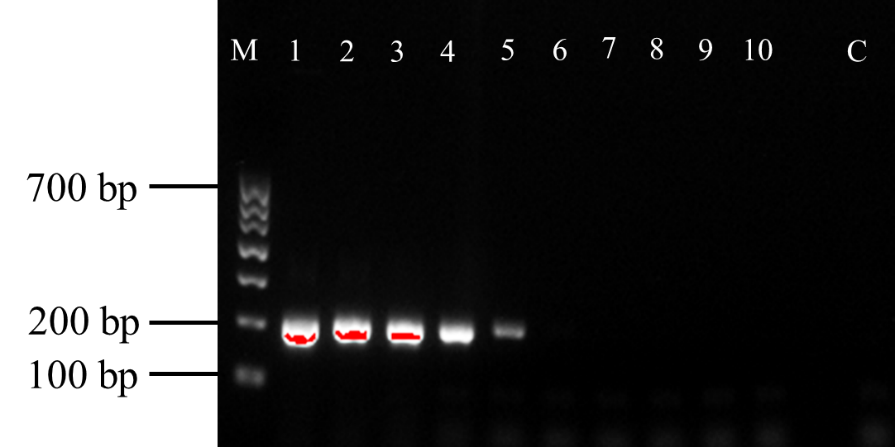


Figure S1. The results of agarose gel electrophoresis of *clfA* target gene amplified by PCR at each dilution.


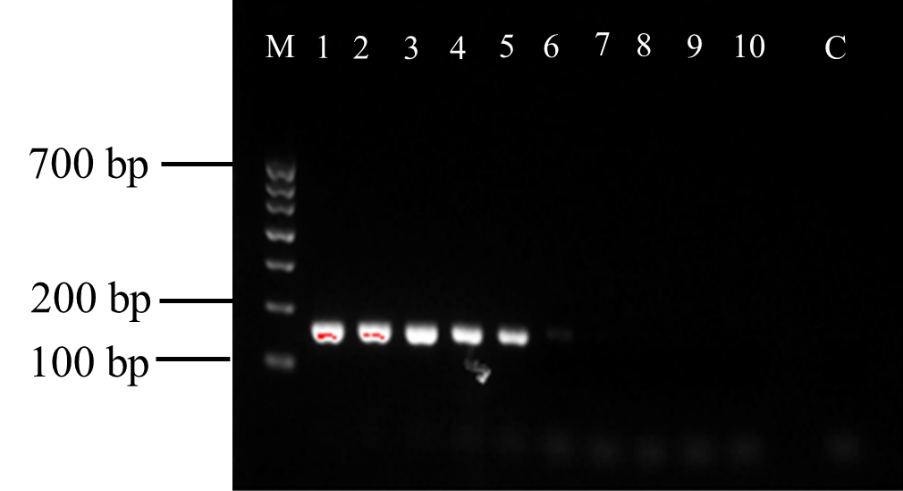


Figure S2. The results of agarose gel electrophoresis of *mecA* target gene amplified by PCR at each dilution.


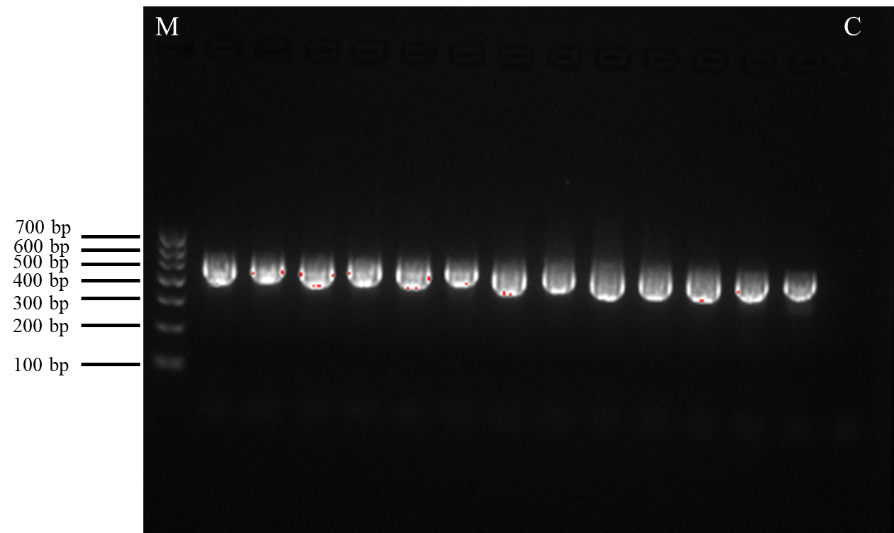


Figure S3. The results of agarose gel electrophoresis of *mecA* gene in clinical samples after PCR amplification
